# Beyond Tuberculosis: Late Diagnosis of Cystic Fibrosis in an Adolescent with Bronchiectasis and Recurrent *Pseudomonas aeruginosa* Isolation: A Case Report

**DOI:** 10.3390/diagnostics16193102

**Published:** 2026-09-24

**Authors:** Oana Mariana Mihailov, Valerii Luțenco, Lavinia Țocu, Raul Mihailov, Loredana Stavăr Matei, George Țocu

**Affiliations:** 1 Faculty of Medicine and Pharmacy, Research Center in the Medical-Pharmaceutical Field, “Dunărea de Jos” University, 800008 Galati, Romania; oana.mihailov@ugal.ro (O.M.M.); valerii.lutenco@ugal.ro (V.L.); lavinia.tocu@ugal.ro (L.Ț.); george.tocu@ugal.ro (G.Ț.); 2 “Sfântul Spiridon” Clinical Hospital of Pneumophthisiology, 800552 Galati, Romania; 3 “Sf. Apostol Andrei” County Emergency Clinical Hospital, 800578 Galati, Romania; 4 “Sf. Ioan” Children’s Emergency Hospital, 800487 Galati, Romania

**Keywords:** cystic fibrosis, bronchiectasis, pulmonary tuberculosis, *Pseudomonas aeruginosa*, *Klebsiella* spp., adolescent, sweat chloride, inhaled tobramycin, late diagnosis

## Abstract

**Background and Clinical Significance:** Cystic fibrosis may remain undiagnosed until adolescence, when recurrent respiratory infections and bronchiectasis are attributed to a previous severe infection. **Case Presentation:** We report the case of a male adolescent in whom approximately five years elapsed between microbiologically confirmed pulmonary tuberculosis in July 2021 and recognition of cystic fibrosis in August 2026. Archived records documented two sputum specimens that were acid-fast bacilli smear-positive (1+) and culture-positive (1+) for *Mycobacterium tuberculosis* complex. During adolescence, persistent bronchiectasis, recurrent Gram-negative respiratory infections, digital clubbing, and repeated *Pseudomonas aeruginosa* isolation prompted etiologic reassessment. A single documented sweat chloride concentration was 114 mmol/L. *CFTR* analysis of blood collected on 18 August 2026, reported on 16 September 2026, identified homozygous c.1521_1523delCTT (p.Phe508del; F508del), providing molecular confirmation. Spirometry showed FEV_1_ 2.69 L (79.6% predicted), FVC 3.73 L (91.56% predicted), and FEV_1_/FVC 72.01% (z-score −2.58), consistent with airflow obstruction; without total lung capacity measurements, restriction or a mixed ventilatory defect could not be confirmed. The previously recorded azithromycin regimen was 500 mg orally three times weekly. Tobramycin inhalation solution 300 mg/5 mL was prescribed as one ampoule twice daily for 28 days, followed by 28 days off, with three planned cycles and salbutamol 15 min before administration. Management of cystic fibrosis also included airway-clearance therapy, dornase alfa 2.5 mg once daily, a high-calorie diet, pancreatic enzyme replacement, and vitamin supplementation. Longitudinal post-treatment spirometry, anthropometry, and validated quality-of-life measurements were not available. **Conclusions:** Previous pulmonary tuberculosis should not terminate etiologic investigation when bronchiectasis, repeated *Pseudomonas aeruginosa* isolation, digital clubbing, or progressive structural lung disease persists. Earlier recognition may permit earlier coordinated cystic fibrosis care and genotype-appropriate CFTR-modulator assessment.

## 1. Introduction

Cystic fibrosis (CF) is an autosomal recessive disorder caused by pathogenic variants in the *cystic fibrosis transmembrane conductance regulator (CFTR)* gene. Abnormal CFTR-mediated ion transport results in altered airway surface liquid, impaired mucociliary clearance, chronic airway inflammation, recurrent infection, and progressive structural lung damage [1,2,3]. Although neonatal screening and expanded molecular testing have improved early recognition, delayed diagnosis remains possible, particularly when the respiratory phenotype is initially attributed to another condition [1,2].

Bronchiectasis in children and adolescents has a broad differential diagnosis, including severe or recurrent infection, pulmonary tuberculosis (TB), primary immunodeficiency, primary ciliary dyskinesia, aspiration, and cystic fibrosis. Current pediatric bronchiectasis guidelines emphasize the importance of identifying an underlying cause because etiologic treatment can alter the subsequent trajectory [4,5,6]. A history of pulmonary TB may provide a credible explanation for structural lung disease, but it does not exclude a concurrent disorder of mucociliary clearance.

*Pseudomonas aeruginosa* is a major pathogen in cystic fibrosis lung disease. Repeated isolation is associated with chronic airway inflammation, structural damage, and accelerated decline in lung function, making microbiological surveillance and appropriate antimicrobial strategies central components of care [7,8,9,10]. In this case, pulmonary tuberculosis was diagnosed in July 2021, and cystic fibrosis was clinically recognized by 18 August 2026, an interval of approximately five years and one month; molecular confirmation followed on 16 September 2026. The case is clinically instructive because genuine, microbiologically confirmed TB coexisted with an inherited disorder, illustrating the risk of premature diagnostic closure.

## 2. Case Presentation

### 2.1. Patient Information and Previous History

A male adolescent followed in a pediatric pulmonology service was clinically diagnosed with cystic fibrosis at 15 years of age. Identifying information has been removed from this manuscript. Available family history indicated a healthy mother, a father with an atopic background, and a paternal grandfather with a history of tuberculosis. Pregnancy and delivery history was obtained from the mother and was not supported by contemporaneous obstetric documentation; the child was reportedly born at term with a birth weight of approximately 3000 g. Although records from the 2021 tuberculosis episode were not available at the initial reassessment, archived hospital laboratory reports were subsequently retrieved.

At 10 years of age, pulmonary TB was microbiologically confirmed. Two sputum specimens collected on 5 July 2021 were acid-fast bacilli smear-positive (1+) and culture-positive (1+), with identification of the *Mycobacterium tuberculosis* complex. Phenotypic testing reported susceptibility to rifampicin and isoniazid at the concentrations used by the laboratory. Follow-up sputum specimens collected on 3 and 6 September 2021 were smear- and culture-negative. The specialist history recorded completion of a six-month anti-TB treatment course. Thereafter, persistent respiratory abnormalities and bronchiectasis were considered largely in the context of previous TB. Post-TB bronchiectasis is a recognized sequela of pulmonary TB and may result from parenchymal destruction, bronchial injury, and traction bronchiectasis, often with upper-lobe predominance [11,12,13,14,15].

At approximately 14 years of age, recurrent lower respiratory tract infections and a reported weight loss of approximately 10 kg led to renewed specialist evaluation. Two sputum specimens collected on 28 October 2025 and bronchial aspirate and bronchoalveolar lavage specimens collected on 6 November 2025 were negative by acid-fast bacilli microscopy and culture, arguing against active pulmonary TB at that reassessment without negating the confirmed 2021 episode. Chest CT during the November 2025 admission, as summarized in the specialist discharge documentation, showed multiple cylindrical bronchiectases with air-filled lumina, distributed from the hilar regions towards the periphery in both upper lobes. Associated findings included bilateral interstitial pulmonary micronodules and mediastinal (including subcarinal) and hilar lymphadenopathy measuring approximately 13 mm. No pleural or pericardial fluid collections were reported. In November 2025, *Klebsiella* spp. and *Pseudomonas aeruginosa* were recovered from respiratory specimens in the setting of pneumonia/bronchopneumonia.

Bronchoscopy demonstrated a patent right bronchial tree, with moderate purulent secretions and inflamed bronchial mucosa, consistent with chronic suppurative airway disease. The persistence of these respiratory abnormalities, despite previous treatment, further supported the need for continued clinical and etiologic reassessment.

### 2.2. Clinical and Microbiological Timeline

The main clinical, microbiological, and diagnostic milestones are summarized in Table 1. The approximately five-year interval refers to the time between the confirmed tuberculosis diagnosis in July 2021 and recognition of cystic fibrosis by August 2026; it does not establish the onset date of CF manifestations. The timeline distinguishes microbiological isolation from clinically diagnosed infection and does not use the term ‘chronic *Pseudomonas aeruginosa* infection’ because the available sampling history does not permit confirmation of a prespecified accepted definition.

### 2.3. Anti-Infective Treatment

During clinically documented infectious episodes, ciprofloxacin was administered at 500 mg every 12 h for 14 days when an oral antipseudomonal option was appropriate and susceptibility was documented. Intravenous ceftriaxone was administered at 1 g every 12 h for 10 days for episodes associated with susceptible *Klebsiella* spp.; it should not be interpreted as Pseudomonas-directed therapy. The previously recorded azithromycin regimen was 500 mg orally three times weekly (Monday, Wednesday, and Friday) as adjunctive long-term maintenance treatment rather than sole eradication therapy for *Pseudomonas aeruginosa*; however, its specific indication and duration were not stated in the August 2026 specialist prescriptions. In August 2026, tobramycin inhalation solution 300 mg/5 mL was prescribed as one ampoule by nebulization twice daily, morning and evening, for 28 days, followed by 28 days off treatment; three cycles were planned. Salbutamol nebulization was recommended 15 min before tobramycin. This was cyclic antipseudomonal treatment in the setting of recurrent *Pseudomonas aeruginosa*-associated respiratory disease; the available records do not establish whether the intended objective was eradication or suppression, do not confirm completion of all planned cycles, and do not document a formal chronic-infection classification [7,8,16].

In February–March 2026, during an episode documented as *Pseudomonas aeruginosa* pneumonia, the patient received ciprofloxacin, symptomatic measures, respiratory physiotherapy, and home inhaled TOBI every 12 h for one month, followed by one month off, for two cycles according to the specialist history. Digital clubbing and thoracic kyphosis were noted. Subsequent respiratory cultures again yielded *Pseudomonas aeruginosa*, with changes in susceptibility to aminoglycosides and fluoroquinolones. This historical regimen is distinct from the August 2026 prescription described above.

### 2.4. Establishment of the Cystic Fibrosis Diagnosis

At 15 years of age, persistent bronchiectasis, recurrent Gram-negative respiratory infections, repeated *Pseudomonas aeruginosa* isolation, and digital clubbing prompted reconsideration of the underlying etiology. One sweat chloride result of 114 mmol/L was documented in the available specialist letter; the original test report, precise test date, method, and a repeat measurement were not available. In the compatible clinical setting, this value strongly supported CF [1,2,3]. CFTR analysis of EDTA blood collected on 18 August 2026 identified the pathogenic c.1521_1523delCTT variant in the homozygous state (p.Phe508del; F508del); the report was issued on 16 September 2026 and provided molecular confirmation. The additional c.1210-12T [9] finding was classified by the laboratory as a polymorphism and is not presented as a second disease-causing variant.

At the specialist assessment on 18 August 2026, spirometry showed FEV_1_ 2.69 L (79.6% predicted), FVC 3.73 L (91.56% predicted), and FEV_1_/FVC 72.01% (z-score −2.58), consistent with airflow obstruction. In the absence of total lung capacity measurements, a restrictive component or a mixed ventilatory defect could not be confirmed. No bronchodilator response was documented. The patient weighed 52.5 kg and was 168 cm tall, corresponding to a calculated BMI of approximately 18.6 kg/m^2^; the specialist record described intestinal malabsorption, mild protein-energy malnutrition, and a history of approximately 10 kg weight loss. He was afebrile and in generally good condition, with a productive cough, digital clubbing, thoracic kyphosis, and bilateral crackles.

### 2.5. Radiological Evolution

Serial posteroanterior chest radiographs were available from July 2022, October 2025, February 2026, and July 2026 (Figure 1). Across the series, the dominant pattern was one of chronic bilateral perihilar and upper-zone structural lung abnormality, with coarse peribronchial/interstitial markings and bronchiectatic-type changes. The abnormalities were consistently more conspicuous in the upper lobes and perihilar regions. No pleural effusion was evident on the provided studies.

The July 2022 radiograph already showed persistent right-predominant upper-lobe/perihilar parenchymal and peribronchial abnormalities, compatible with chronic postinfectious structural change in the clinical context. By 15 October 2025, there was a clear increase in bilateral patchy and reticulonodular/peribronchial opacity, particularly in the upper and perihilar zones, consistent with an acute infectious or inflammatory exacerbation superimposed on chronic lung disease. The 26 October 2025 study showed partial interval clearing of the multifocal opacities, while chronic bilateral perihilar and upper-zone abnormalities persisted.

On 17 February 2026, recurrent right upper-lobe and perihilar patchy opacity was again present on a background of bilateral chronic bronchiectatic/interstitial change, temporally corresponding to the subsequent period in which *Pseudomonas aeruginosa* pneumonia was documented. The 8 July 2026 radiograph demonstrated partial improvement of the superimposed opacities but persistent structural abnormalities, supporting a chronic suppurative airway phenotype rather than complete radiographic resolution after isolated infectious episodes. The radiographic pattern itself is not specific for cystic fibrosis and cannot reliably distinguish post-TB sequelae from CF-related bronchiectasis. Therefore, the radiographic findings should be interpreted together with computed tomography, microbiological results, and the longitudinal clinical course. In this case, their main value lies in the persistence and recurrence of abnormalities despite treatment.

### 2.6. CF-Directed Treatment

The specialist treatment plan after CF diagnosis included a high-calorie diet; pancreatic enzyme replacement with Kreon 25,000, prescribed as 12 capsules daily divided between meals and snacks, in the context of documented intestinal malabsorption and mild protein-energy malnutrition; dornase alfa 2.5 mg by nebulization once daily as long-term therapy; tobramycin inhalation solution 300 mg/5 mL, one ampoule twice daily for 28 days followed by 28 days off, with three planned cycles; salbutamol 15 min before inhaled tobramycin; regular airway-clearance techniques and respiratory physiotherapy; oral rehydration salts; and CF-specific vitamin supplementation. The available records did not include fecal elastase or another objective test confirming exocrine pancreatic insufficiency; pancreatic enzyme replacement is therefore reported as specialist-directed treatment for suspected pancreatic maldigestion, not as proof of laboratory-confirmed exocrine pancreatic insufficiency.

The values recorded on 18 August 2026 represent the initial specialist assessment and not a demonstrated response to the newly prescribed CF-directed regimen. The discharge status was described as improved, but no quantified symptom change, repeat spirometry, serial weight or BMI, validated quality-of-life score, or longitudinal patient-reported assessment was available. Consequently, sustained pulmonary, nutritional, or quality-of-life benefit could not be assessed. Reassessment was planned after three months, or earlier if the genetic result supported consideration of CFTR-modulator therapy.

Care involved the local pediatric pneumology service, a specialist pediatric service in Bucharest, and the medical genetics laboratory/service. Respiratory physiotherapy was documented, and nutritional management formed part of the specialist recommendations. Separate consultations with a dietitian, psychologist, or a formally constituted multidisciplinary CF team were not documented; we therefore report the verified inter-service involvement without inferring participation by unrecorded professionals.

This strategy is consistent with the major principles of contemporary CF care: regular airway clearance [17], management of *Pseudomonas aeruginosa* [7,8], use of dornase alfa in appropriate patients [18], optimization of nutritional status [19,20], and assessment for CFTR-modulator eligibility [21,22]. Pancreatic function, fat-soluble vitamin status, bone health, and longitudinal growth should also be assessed systematically [19,20,23].

## 3. Discussion

The central diagnostic challenge in this case was the coexistence of two plausible explanations for chronic lung disease: bacteriologically confirmed pulmonary TB during childhood and an inherited disorder of mucociliary clearance that was recognized later. TB can produce permanent bronchial and parenchymal injury, including bronchiectasis, and respiratory sequelae may persist after completion of appropriate therapy [11,12,13,14,15]. Therefore, attributing the bronchiectasis to previous TB was initially reasonable. However, the subsequent clinical trajectory was not fully explained by that history.

The radiographic evolution reinforces this diagnostic ambiguity. The 2022 film was compatible with chronic postinfectious change, whereas the later studies showed recurrent bilateral upper-lobe/perihilar inflammatory opacities superimposed on persistent structural disease. This pattern is compatible with repeated infectious exacerbations in bronchiectatic lungs but is not etiologically specific. In retrospect, the failure of the structural abnormalities to resolve and the recurrence of superimposed opacities provided an additional reason to revisit the initial post-TB attribution and investigate an underlying mucociliary-clearance disorder.

Clinically documented Gram-negative respiratory infections involving *Klebsiella* spp., together with repeated *Pseudomonas aeruginosa* isolation and later co-isolation of both organisms, represented important diagnostic signals. The term isolation is used for recovery from a respiratory specimen, whereas infection is reserved for episodes interpreted in conjunction with clinical findings. Because the available sampling history did not permit confirmation of an accepted definition of chronic infection, the patient-specific term ‘chronic *Pseudomonas aeruginosa* infection’ is avoided. Repeated *Pseudomonas aeruginosa* isolation in an adolescent with extensive bronchiectasis should prompt systematic investigation for an underlying disorder such as CF, even when another cause of bronchiectasis has already been identified [9,10].

The serial susceptibility profiles are also clinically informative. *Pseudomonas aeruginosa* remained susceptible to several antipseudomonal agents, but susceptibility to amikacin and fluoroquinolones varied over time. This reinforces the importance of repeated respiratory cultures and susceptibility-guided antimicrobial treatment. CF guidelines recommend prompt eradication of initial *Pseudomonas aeruginosa* infection and, in chronic infection, longer-term suppressive strategies, including inhaled antibiotics [7,8].

The repeated use of ciprofloxacin in this case is notable because it represents an oral antipseudomonal option when susceptibility is documented, and clinical severity permits oral treatment. Ceftriaxone, by contrast, is not a standard antipseudomonal agent and should not be interpreted as treatment directed against *Pseudomonas aeruginosa*. In this case, its use should be linked to the individual clinical episodes and organisms for which it was selected, including susceptible *Klebsiella* spp. This distinction improves the microbiological accuracy of the case narrative.

Azithromycin 500 mg orally three times weekly (Monday, Wednesday, and Friday) was recorded in the earlier treatment history as adjunctive long-term maintenance therapy rather than sole eradication therapy for *Pseudomonas aeruginosa*. This regimen is consistent with an anti-inflammatory and immunomodulatory role, with the potential to reduce pulmonary exacerbations in the context of CF-associated bronchiectasis and recurrent *Pseudomonas aeruginosa* isolation [24,25,26]. However, azithromycin was not listed in the August 2026 specialist prescriptions available for this revision, and its exact indication and duration could not be independently verified from those documents.

Intermittent inhaled tobramycin in 28-day cycles is supported by randomized clinical evidence and CF guidance [7,8,16]. In August 2026, tobramycin inhalation solution 300 mg/5 mL was prescribed as one ampoule twice daily for 28 days, followed by 28 days off, with three planned cycles and salbutamol 15 min before administration. This was cyclic antipseudomonal treatment after recurrent *Pseudomonas aeruginosa* isolation; the available record does not establish whether the intended therapeutic objective was eradication or suppression, does not document completion of all cycles, and does not establish a prespecified definition of chronic infection.

The introduction of dornase alfa and structured airway clearance is physiologically coherent with CF lung disease. Dornase alfa reduces sputum viscoelasticity by cleaving extracellular deoxyribonucleic acid and can improve pulmonary outcomes in appropriate patients [18,27]. Airway-clearance techniques should be performed regularly and individualized according to age, disease burden, adherence, and patient preference [17,28].

Nutritional management is another essential component of CF care. Current guidance emphasizes systematic nutritional assessment, adequate energy intake, pancreatic enzyme replacement when exocrine pancreatic insufficiency is documented, and supplementation of fat-soluble vitamins [19,20,23]. At the specialist assessment, BMI was approximately 18.6 kg/m^2^, intestinal malabsorption and mild protein-energy malnutrition were recorded, and a history of approximately 10 kg weight loss was noted. Kreon 25,000 was prescribed as 12 capsules daily, divided between meals and snacks. The available records did not include serial post-treatment anthropometry, fecal elastase, or other objective pancreatic-function testing; therefore, nutritional recovery and laboratory-confirmed exocrine pancreatic insufficiency could not be quantified from the supplied data.

From a diagnostic perspective, the case illustrates a broader principle: bronchiectasis is a phenotype, not an etiologic diagnosis. In a child or adolescent with extensive bronchiectasis, recurrent infection, *Pseudomonas aeruginosa*, digital clubbing, or an unexpectedly persistent disease course, the underlying diagnosis should be periodically revisited [4,5,6]. A previous infection, including TB, should not close the diagnostic pathway.

The potential therapeutic consequences of delayed diagnosis are substantial. CFTR-modulator therapies can improve lung function and reduce pulmonary exacerbations in eligible patients [21,22]. Molecular analysis confirmed homozygous F508del in this patient. Earlier recognition could therefore have enabled earlier assessment for genotype-appropriate modulator therapy, subject to age, eligibility, access, and clinical suitability at the relevant time. However, the records do not document actual initiation of a CFTR modulator or a subsequent response, and the magnitude of any retrospective benefit cannot be established.

## 4. Strengths and Limitations

The main strengths of this report are the longitudinal microbiological record, documenting recurrent Gram-negative airway infections involving *Klebsiella* spp. and *Pseudomonas aeruginosa*, repeated *Pseudomonas aeruginosa* isolation, subsequent co-isolation, and evolving antimicrobial susceptibility profiles, together with the availability of serial chest radiographs spanning 2022–2026. The case also provides a clinically important diagnostic contrast between a plausible post-TB explanation and an ultimately demonstrated CF phenotype.

The principal limitations are those inherent to a retrospective single-patient report: incomplete historical documentation, absence of a comparator, uncertainty regarding the precise onset of CF manifestations, only one documented sweat chloride result without an available repeat test report, incomplete cross-sectional imaging documentation, and lack of quantitative longitudinal microbiological data. Although molecular testing subsequently confirmed homozygous F508del, post-diagnostic follow-up was insufficient to assess sustained response: serial spirometry, anthropometry, validated quality-of-life measurements, and detailed patient-reported outcomes were unavailable. Objective evidence of pancreatic exocrine insufficiency and documentation of a formally constituted multidisciplinary CF team were also not available. These limitations preclude causal attribution and restrict generalizability.

## 5. Conclusions

This case illustrates the risk of assigning a single definitive etiology to bronchiectasis in a child or adolescent with a significant previous infection. Pulmonary TB was microbiologically confirmed in July 2021, but persistent bronchiectasis, clinically documented Gram-negative respiratory infections, repeated *Pseudomonas aeruginosa* isolation, digital clubbing, and ongoing abnormalities justified renewed etiologic investigation. Approximately five years later, a sweat chloride concentration of 114 mmol/L supported the diagnosis of cystic fibrosis, and CFTR analysis subsequently confirmed homozygous c.1521_1523delCTT (p.Phe508del; F508del).

The key clinical message is that previous TB does not exclude cystic fibrosis. Etiologic assessment should be revisited when bronchiectasis and recurrent respiratory disease persist, particularly when *Pseudomonas aeruginosa* is repeatedly isolated. Earlier recognition could enable earlier airway-clearance therapy, inhaled antimicrobial treatment, nutritional intervention, coordinated specialist care, and assessment for genotype-appropriate CFTR-modulator therapy. In this patient, any benefit of earlier modulator initiation remains a potential opportunity rather than an observed outcome.

## Figures and Tables

**Figure 1:**
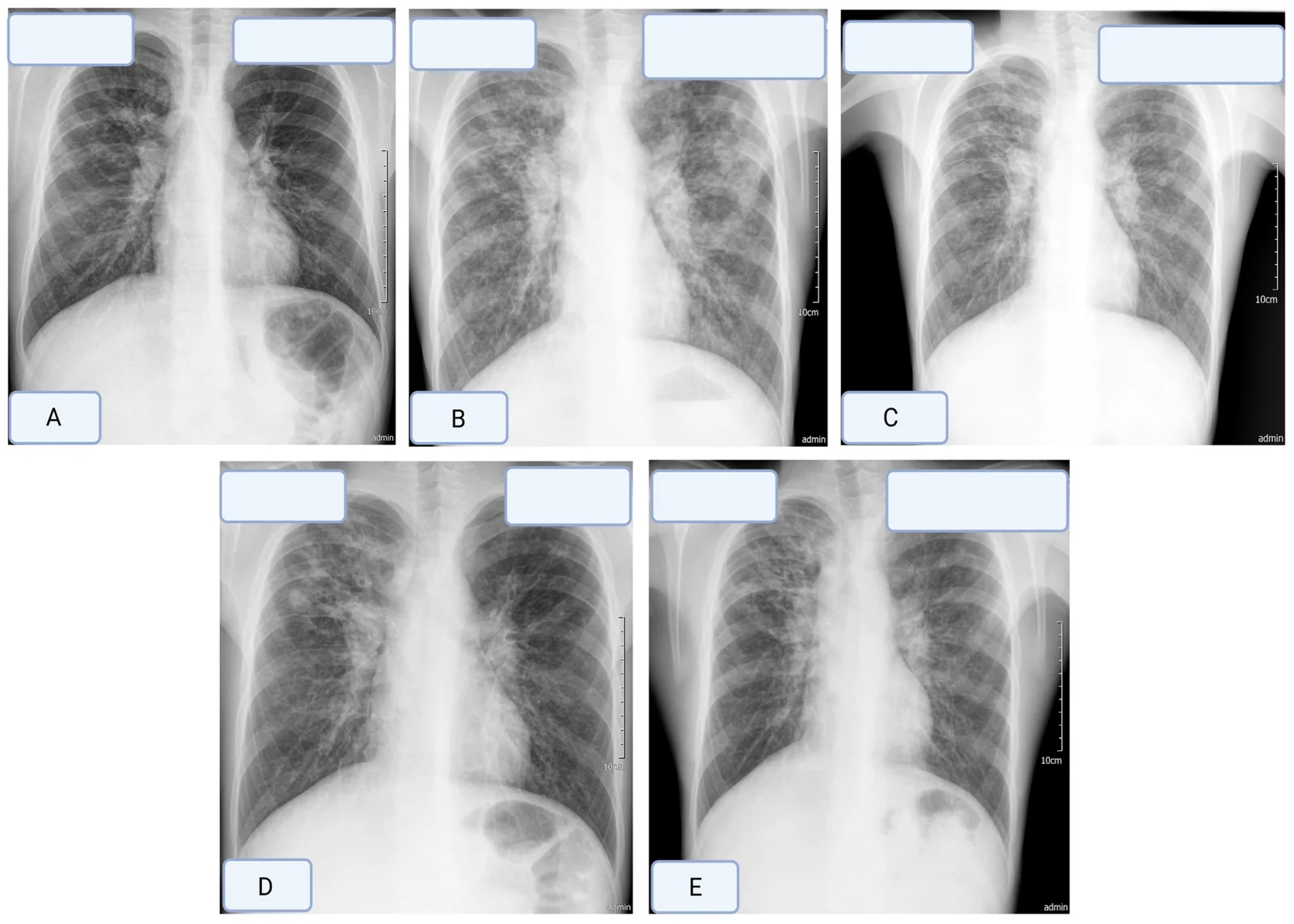
Serial posteroanterior chest radiographs illustrating the longitudinal pulmonary evolution: (**A**) 12 July 2022, showing chronic right-predominant upper-lobe/perihilar structural and peribronchial abnormalities; (**B**) 15 October 2025, showing increased bilateral patchy and reticulonodular/peribronchial opacities, most evident in the upper and perihilar regions; (**C**) 26 October 2025, showing partial interval improvement of the superimposed opacities with persistent chronic abnormalities; (**D**) 17 February 2026, showing recurrent right upper-lobe/perihilar patchy opacity on a background of bilateral chronic bronchiectatic/interstitial changes; and (**E**) 8 July 2026, showing partial clearing of acute opacities with persistent bilateral structural lung disease. Patient-identifying information was redacted for publication.

**Table 1:** Clinical, microbiological, and diagnostic timeline of the patient.

Age/Date	Clinical, Microbiological, or Diagnostic Event	Clinical Relevance
10 years, 5 July 2021	Two sputum specimens were AFB smear-positive (1+) and culture-positive (1+); *Mycobacterium tuberculosis* complex was identified.	Microbiologically confirmed pulmonary TB.
10 years, 3 and 6 September 2021	Follow-up sputum specimens were AFB smear-negative and culture-negative.	Documented microbiological conversion during follow-up.
14 years, 28 October and 6 November 2025	Two sputum specimens, bronchial aspirate, and bronchoalveolar lavage were AFB microscopy-negative and culture-negative.	No microbiological evidence of active TB at the later bronchiectasis reassessment.
14 years, November 2025	*Klebsiella* spp. recovered from respiratory specimens in the setting of pneumonia/bronchopneumonia.	Persistent suppurative airway disease prompted continued surveillance.
14 years, November 2025	*Pseudomonas aeruginosa* isolated from sputum; antipseudomonal susceptibility documented.	First documented *Pseudomonas aeruginosa* isolation in the available retrospective record.
15 years, February–March 2026	*Pseudomonas aeruginosa* pneumonia; digital clubbing and thoracic kyphosis documented.	Clinical warning signs for chronic suppurative lung disease and possible CF.
15 years, February 2026	*Pseudomonas aeruginosa* susceptible to ceftazidime, ciprofloxacin, gentamicin, piperacillin/tazobactam, and carbapenems; resistant to amikacin and levofloxacin.	Evolving antimicrobial susceptibility informed treatment.
15 years, July 2026	*Klebsiella* spp. isolated from sputum with a reported antimicrobial susceptibility profile.	Recurrent Gram-negative respiratory infection.
15 years, August 2026	*Pseudomonas aeruginosa* with associated *Klebsiella* spp. recovered from respiratory specimens.	Repeated *Pseudomonas aeruginosa* isolation with co-isolation of *Klebsiella* spp.
15 years, by 18 August 2026	A documented sweat chloride concentration was 114 mmol/L. Spirometry showed FEV_1_ 2.69 L (79.6% predicted), FVC 3.73 L (91.56% predicted), and FEV_1_/FVC 72.01% (z-score −2.58).	Clinical recognition of CF; airflow obstruction was supported by the reduced FEV_1_/FVC ratio. Restriction or a mixed defect could not be confirmed without total lung capacity measurement.
15 years, 16 September 2026	CFTR analysis identified homozygous c.1521_1523delCTT (p.Phe508del; F508del).	Molecular confirmation of cystic fibrosis.

Note: Antimicrobial susceptibility results presented in Table 1 were transcribed from the available laboratory reports and should be interpreted in the context of the testing methods used, contemporaneous interpretive breakpoints, specimen quality, clinical syndrome, and prior antimicrobial exposure. Abbreviations: AFB, acid-fast bacilli; CF, cystic fibrosis; CFTR, cystic fibrosis transmembrane conductance regulator; FEV_1_, forced expiratory volume in 1 s; FVC, forced vital capacity; TB, tuberculosis.

## Data Availability

The original contributions presented in this study are included in the article. Additional patient-level data are not publicly available due to patient privacy considerations. Further inquiries can be directed to the corresponding authors.
